# The Association Between Physical Activity and Domain‐Specific Cognitive Function in the Elderly: A Cross‐Sectional Study and Genetic Analysis

**DOI:** 10.1002/brb3.71423

**Published:** 2026-05-05

**Authors:** Xingxiao Yin, Hao Peng, Yanqi Li, Yanping Song, Longjiang Chen, Na Yao, Pengcheng Li, Zhijuan He, Hongbo Chen, Li Huang, Zhen Shen, Qigang Chen

**Affiliations:** ^1^ School of Physical Education Yunnan Normal University Kunming Yunnan China; ^2^ Department of Rehabilitation Medicine Third Affiliated Hospital of Yunnan University of Chinese Medicine Kunming Yunnan China

**Keywords:** cognitive function, Mendelian randomization, NHANES, physical activity, regular activity, weekend warrior

## Abstract

**Background:**

Population aging is intensifying worldwide, increasing the prevalence of dementia. More than 50 million people globally are affected by dementia. There is some evidence that physical activity (PA) benefits cognitive function (CF). However, it is unclear which types and amounts of PA are best for specific cognitive domains.

**Methods:**

This study used a cross‐sectional design and genetic analysis to test its hypotheses. Participants were drawn from the National Health and Nutrition Examination Survey (NHANES) 2011–2014, including 2578 adults aged 60 years or older. Cognitive abilities were measured using standardized tests, and PA levels were assessed with the Global Physical Activity Questionnaire (GPAQ). The analysis included multivariable regression, threshold effect testing, and subgroup analysis to explore links between PA patterns, intensities, and specific CF domains. Furthermore, a Mendelian randomization (MR) approach was employed to assess whether PA intensity exerts an effect on CF.

**Results:**

NHANES data analysis showed that the regular activity (RA) pattern was positively associated with the Consortium to Establish a Registry for Alzheimer's Disease Word Learning test (CERAD‐WL) scores (*β* = 0.07; 95% confidence interval [CI]: 0.02, 0.16; *p* = 0.018), Animal Fluency Test (AFT) scores (*β* = 0.31; 95% CI: 0.21, 0.41; *p* = 0.002), Digit Symbol Substitution Test (DSST) scores (*β* = 0.19; 95% CI: 0.09, 0.30; *p* = 0.017), and overall CF (*β* = 0.23; 95% CI: 0.14, 0.32; *p* < 0.004). Threshold‐effect analysis revealed an inverted U‐shaped relationship between PA levels and cognitive performance. The inflection points occurred at 650, 535, and 550 min per week for AFT, DSST, and overall CF, respectively. Above these values, further cognitive gains plateaued. The inverse‐variance weighted (IVW) method of the MR analyses showed that moderate‐intensity physical activity (MPA) increased correct counting (odds ratio [OR] = 0.710; 95% CI: 0.606, 0.832; *p* < 0.001), fluid intelligence (OR = 0.470; 95% CI: 0.346, 0.638; *p* < 0.001), and overall cognitive performance (OR = 0.707; 95% CI: 0.653, 0.764; *p* < 0.001). For vigorous‐intensity physical activity (VPA), we observed causal associations with memory outcomes, reaction time, correct matches, fluid intelligence, and cognitive performance.

**Conclusion:**

The findings suggest that PA is associated with multiple domains of CF, and the RA pattern is linked to better cognitive performance. Genetically, VPA appears to have a stronger promotive effect. Thus, different patterns and intensities of PA may help delay cognitive decline and maintain brain health in older adults.

## Graphical abstract

Graphical abstract of the relationship between physical activity and cognitive function in older adults. Regular activity was associated with better cognitive performance, and vigorous‐intensity physical activity showed stronger genetic benefits across multiple cognitive domains.

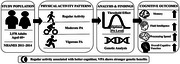

## 1 Background

With the rapid global demographic shift, cognitive health problems have become a major public health challenge in older adults. Cognitive decline progresses from subjective cognitive impairment (SCI) to mild cognitive impairment (MCI) and, eventually, to dementia (Pike et al. 2022). It is not a one‐dimensional issue but a far‐reaching disruption affecting multiple domains, including complex attention, executive function, learning and memory, and social cognition (Salthouse 2012). Currently, more than 55 million people worldwide live with dementia, a figure expected to double in the next few decades (Chowdhary et al. 2022; Vellas et al. 2018). Recent studies have underscored that cognitive impairment often co‐occurs with other health deficits in older adults, such as Vitamin D deficiency (Guo et al. 2024a), further complicating the clinical landscape. Given the lack of effective pharmacological treatments, identifying modifiable risk factors is crucial for maintaining cognitive health.

Physical activity (PA), defined as energy expenditure through skeletal muscle contraction, is a primary intervention for maintaining brain health. Research indicates that PA improves the quality of life and cognitive function (CF) in older adults (Lamb et al. 2018; Sabia et al. 2017). Beyond its direct impact on cognition, PA is intricately linked to various health behaviors, including sleep quality and the reduction of addictive behaviors like smoking (Zhang et al. 2023a; Zhang et al. 2025), which may indirectly benefit brain health. Biologically, routine exercise is hypothesized to boost CF by enhancing cerebral blood circulation, lowering systemic inflammation, and stimulating neuroplasticity through the upregulation of neurotrophic factors (Frederiksen et al. 2015; Henskens et al. 2018; Nguyen et al. 2023; Zhang et al. 2023b; Zhu et al. 2020). Moreover, the interplay between lifestyle factors and psychological health, such as the association between depression and physiological deficiencies (Mo et al. 2023), suggests that PA's role may be multifaceted. However, most studies focus on global CF and overlook specific domains like executive function and reaction time (Wu et al. 2025). The optimal patterns and intensities of exercise required to target these specific domains remain unclear, limiting the development of customized interventions.

To address these gaps, we utilized data from the 2011–2014 National Health and Nutrition Examination Survey (NHANES) to examine the cross‐sectional relationship between PA patterns and multidimensional cognitive performance. However, observational studies using survey data are often susceptible to recall bias and reverse causation, which can confound causal inference (Luan et al. 2026). To overcome these limitations, we integrated Mendelian randomization (MR) into our framework. MR utilizes genetic variants as instrumental variables (IVs), exploiting the quasi‐random assignment of alleles to reduce confounding and bias (Luo et al. 2025). By combining the large‐scale, real‐world breadth of NHANES with the robust causal architecture of two‐sample MR, this study aims to clarify the causal effects of moderate‐intensity PA (MPA) and vigorous‐intensity PA (VPA) on specific cognitive domains, providing more interpretable and actionable findings for high‐risk populations.

## 2 Materials and Methods

**FIGURE 1:**
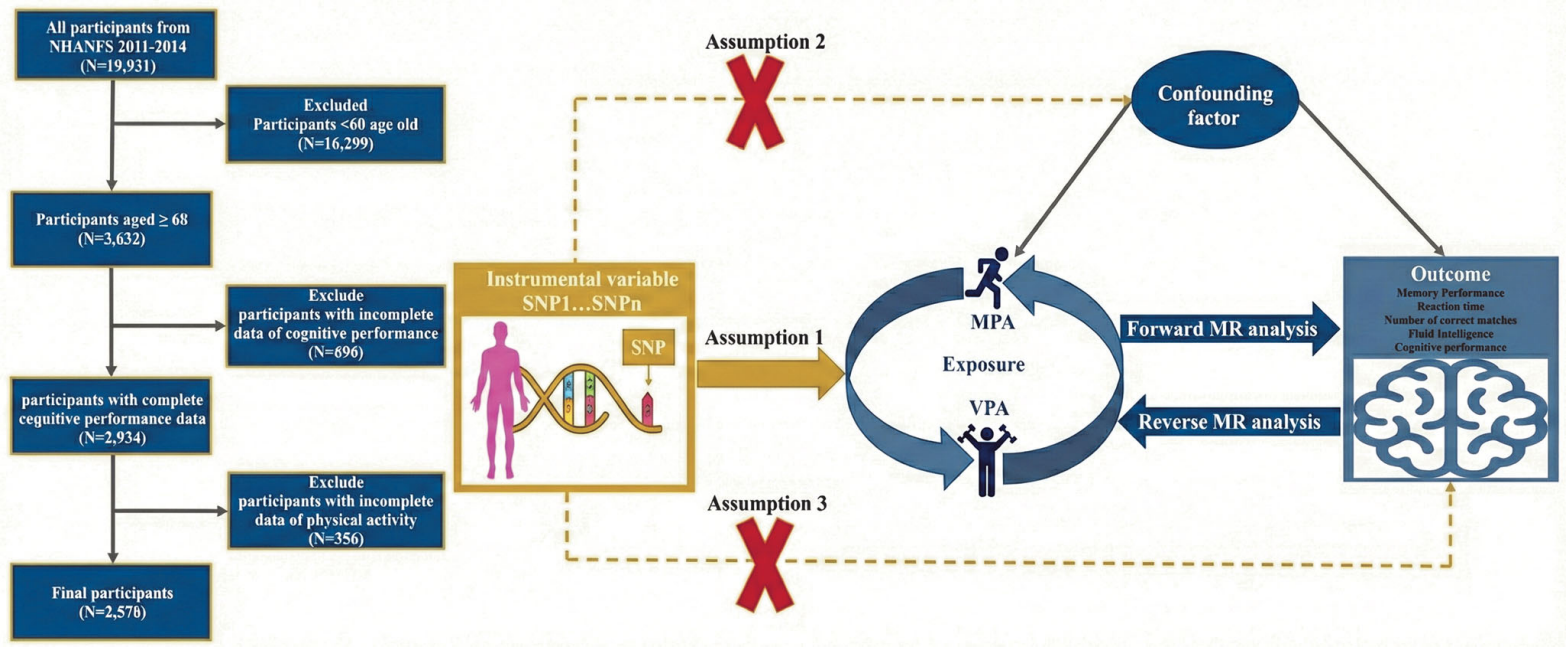
Study design.

As shown in Figure 1, this study created an analytical framework combining cross‐sectional surveys with genetic inference. We first used the NHANES database to examine links between PA patterns, intensity, and multidimensional CF. To overcome the limitations of observational studies, we then applied MR methods to specifically test the impact of MPA and VPA on five core cognitive indicators. This approach combines population and genetic data to examine how PA influences cognition.

### 2.1 Observational Study

#### 2.1.1 Study Population

We used the 2011–2014 NHANES database. Our initial sample had 19,931 people. Since the CF assessment targeted subjects aged 60 or older, we kept only those who met this age requirement. This excluded 16,299 younger individuals. Among the 3632 remaining, we included 2578 older adults with both cognitive test and PA records. We excluded 698 without cognitive tests and 356 without PA records (see Figure 1).

#### 2.1.2 PA

This study used the Global Physical Activity Questionnaire (GPAQ) to measure PA. The questionnaire covered the frequency and duration of MPA and VPA, with sessions of at least 10 min per week (Cleland et al. 2014). US guidelines count 1 min of VPA as 2 min of MPA. We calculated total moderate‐to‐vigorous physical activity (MVPA) by converting VPA to MPA equivalents and adding self‐reported MPA (Li et al. 2024; Piercy et al. 2018). WHO guidelines recommend adults 60+ get 150–300 min of moderate or 75–150 min of vigorous activity per week, or a combination. We analyzed PA as both continuous and grouped variables. Four PA patterns were based on MVPA volume and frequency: inactive (0), insufficient (< 150 min/week), and adequate (≥ 150 min/week), with frequencies of 1–2 sessions per week (weekend warrior [WW]) or ≥ 3 sessions per week (regular activity [RA]) (Bull et al. 2020; Lei et al. 2024). We classified participants into three intensity categories: inactive, MPA‐only, or VPA‐only.

#### 2.1.3 CF

The study used the Consortium to Establish a Registry for Alzheimer's Disease Word Learning test (CERAD‐WL), Animal Fluency Test (AFT), and Digit Symbol Substitution Test (DSST) for cognitive assessment. Specifically, the CERAD‐WL included three immediate memory learning trials, which formed the initial phase before participants completed the AFT and DSST. Following these, a delayed recall trial was administered. In each CERAD‐WL learning trial, participants read 10 unrelated words aloud in random order. After completing the AFT and DSST, they attempted to recall as many CERAD‐WL words as possible. Scores ranged from 0 to 133, based on correct recalls (Morris et al. 1993).

The AFT, administered after the CERAD‐WL immediate recall trials, tests verbal fluency and cognitive control. In this task, people name as many animals as they can in a minute, earning one point for each correct answer. This process measures fluency of verbal retrieval and reaction speed (Brody et al. 2019; Canning et al. 2004). In turn, the DSST follows the AFT and is a paper‐and‐pencil test. It primarily measures information‐processing speed and executive function. The test paper shows nine groups of numbers and symbols at the top. Participants must match symbols to 133 numbers in 2 min, earning one point per correct answer (Amaresha et al. 2014; Campitelli et al. 2023).

To evaluate overall CF across these tests, we constructed a global *Z*‐score in addition to assessing individual cognitive test scores. To make scores comparable and integrable across different cognitive domains, raw scores were standardized (Wilson et al. 2002). We subtracted each individual's cognitive score from the sample mean, then divided the difference by the standard deviation. This calculated a test‐specific *Z*‐score for CERAD‐WL, AFT, and DSST. Lastly, we averaged the three *Z*‐scores to obtain a global *Z*‐score for overall CF. Higher scores in all tests indicate better CF status.

#### 2.1.4 Covariates

Building upon existing research, the study incorporated covariates potentially associated with PA and CF to control for the influence of potential common factors (Dong et al. 2024; Wang et al. 2024a; Wang et al. 2024b)—age (60–69, 70–79, ≥ 80 years), sex (male, female), race (Black non‐Hispanic, White non‐Hispanic, Hispanic/Latino, other), education (less than high school, high school or equivalent, some college or higher), marital status (married or cohabiting, single [widowed/divorced/separated], never married). Body mass index (BMI) was calculated as weight in kilograms divided by height in meters squared and then categorized as underweight (< 18.5 kg/m^2^), normal weight (18.5–24.9 kg/m^2^), overweight (25.0–29.9 kg/m^2^), and obese (≥ 30 kg/m^2^). Household income was measured using the poverty income ratio (PIR) and categorized as low income (PIR < 1.30), medium income (1.30–3.49), and high income (PIR ≥ 3.50). Alcohol consumption was classified into three categories according to the mean number of drinks per day during the last 12 months: light (<= 1 drink/day for women, <= 2 drinks/day for men), moderate (1–3 drinks/day for women, 2–4 drinks/day for men), and heavy (≥ 4 drinks/day for women, ≥ 5 drinks/day for men). Smoking status was self‐reported as a never smoker (less than 100 cigarettes in lifetime), former smoker (more than 100 cigarettes and quit), or current smoker.

Second, as chronic diseases affect cognitive performance, the study also included relevant health conditions: depression, diabetes, high blood pressure, sleep disorders, and a history of stroke. Depression was measured using the Patient Health Questionnaire‐9 (PHQ‐9) with a total score of ≥ 10 defining depressive status (Kroenke et al. 2001). Diabetes was defined by physician diagnosis, current use of hypoglycemic medication, or fasting blood glucose ≥ 7.0 mmol/L. Hypertension was defined as mean systolic blood pressure ≥ 140 mm Hg, mean diastolic blood pressure ≥ 90 mm Hg, or self‐reported use of antihypertensive medication (Dowllah et al. 2023). Sleep disorders were identified by consulting healthcare providers for sleep problems and self‐reported weekday sleep duration of less than 7 h. Stroke history was assessed with questionnaire responses about physician‐diagnosed stroke.

By including these factors, this study could better estimate the link between PA and thinking skills and reduce possible bias from shared causes.

#### 2.1.5 Statistical Analysis

The study followed the rules set by the CDC and NHANES. We used sampling weights throughout. Continuous variables are given as M ± SE, and categorical variables are presented as SE percentages (%) to describe participant baseline characteristics. We assessed the significance of intergroup differences using *t*‐tests or chi‐square tests. Because the dependent variables were continuous, we ran weighted multiple linear regression models to examine associations between PA patterns or intensities and cognitive domains. This produced effect sizes (*β*) and their 95% confidence intervals (CI). We used three stepwise‐adjusted models: Model 1 was unadjusted and included no covariates; Model 2 was adjusted for age, sex, and race; and Model 3 was fully adjusted, including BMI, education, marital status, PIR, smoking status, drinking status, diabetes, hypertension, depressive symptoms, sleep disorders, history of stroke, and all Model 2 covariates. In addition to linear regression, we used restricted cubic splines to identify possible nonlinear relationships. For detected nonlinearities, we applied piecewise linear regression and likelihood ratio tests to identify the inflection point at which PA could begin influencing CF, suggesting a potential threshold.

We also conducted subgroup analyses using the fully adjusted model. The analyses were stratified by age, sex, BMI, smoking status, diabetes, depression, and sleep disorder to assess whether the associations differed across subgroups. To account for multiple comparisons, the significance threshold for subgroup interaction tests was adjusted using the Bonferroni correction. As seven subgroup factors were tested, the Bonferroni‐corrected significance level was set at *P* for interaction < 0.007 (0.05/7). All statistical analyses were performed using R (version 4.3.2) and the EmpowerStats platform. A two‐sided *p* < 0.05 was considered statistically significant for the main analyses.

### 2.2 MR Study

#### 2.2.1 Study Design

In this study, we use MR analysis to investigate the causal effects of MPA and VPA on a range of cognition‐related health outcomes, including memory performance, reaction time, DSST, fluid intelligence, and cognitive performance. To ensure the inferences we make are valid, the chosen IVs must meet three important criteria. First, the instrument needs to be connected to the exposure variable (either MPA or VPA). Second, the idea is that the instrument shouldn't be linked to factors that can change both the exposure and the result. And third is the idea that the instrument should only work through the exposure to change the result, not by any other way (VanderWeele et al. 2014). To test for reverse causation, a reverse MR was also conducted by treating each CF indicator as an exposure and MPA and VPA as outcomes. All genome‐wide association study (GWAS) summary statistics were obtained from the IEU Open GWAS (https://gwas.mrcieu.ac.uk) and analyzed based on publicly available GWAS.

#### 2.2.2 Data Sources and Genetic Tools

Research data are from the UK Biobank. The UK Biobank is renowned for its high‐quality and large sample size, providing a robust resource for research. As for the PA assessment, the data were categorized into two parts: moderate‐intensity activity (*N* = 440,266) and vigorous‐intensity activity (*N* = 460,376) (Wijndaele et al. 2017). Participants reported their PA habits through a questionnaire, with the question being, “How many days per week do you do MPA or VPA for 10 min or longer?” Five different CFs were considered for each of the following categories: memory performance (*n* = 48,080), reaction time (*n* = 2378), number of correct matches (*n* = 113,106), fluid intelligence (*n* = 149,051), and cognitive performance (*n* = 257,841) (Burns et al. 2018; Jaeger 2018; Lee et al. 2018).

IV screening used strict screening criteria (Yin et al. 2022). The significance threshold for PA‐associated single‐nucleotide polymorphisms (SNPs) is set to *p* < 5 × 10^−^^8^. The linkage disequilibrium (LD) filter uses *R* ^2^ < 0.001 and a genetic distance of 10,000 kb. IV strength was tested using the *F* statistic: *F* = [*R* ^2^ (*N* − *K* − 1)]/[*K* (1 − *R* ^2^)]. Here, *R* ^2^ is the SNPs pair's explained variance for the exposure, *N* is the sample size, and *K* is the number of SNPs. Only SNPs with an *F* statistic > 10 were retained to ensure sufficient IV strength. To further increase IV validity, SNPs with opposite allele direction and palindromes were removed. The GWAS Catalog was used to obtain the phenotypes associated with each SNPs. Variants significantly associated with the outcome or potential confounders, such as smoking, drinking, and triglycerides, were removed. After quality control, we achieved a high degree of independence and a sufficient number of SNPs. These can be used as a genetic tool for MR analysis.

#### 2.2.3 Statistical Analysis

The study mainly used the inverse‐variance weighted (IVW) method with random effects to estimate causal effects. This method combines the association effects and the standard errors of each genetic instrument with the exposure factor using IVW. As a result, it derives the overall causal effect with great statistical power (Zhou et al. 2024). We also perform a consistency check using MR‐Egger regression and the weighted median. This improves our inference. To address potential bias from weak IVs, we used the MR robust adjusted profile score (MR‐RAPS) method, which corrects for parameter bias and improves estimation precision (Zhao et al. 2018). Results are expressed as odds ratios (OR) with their 95% CI. The significance level is set at *p* < 0.05. We assessed heterogeneity using Cochran's *Q* test to assess discrepancies between MR‐Egger and IVW results. Horizontal pleiotropy was assessed using the MR‐Egger intercept test and the MR‐PRESSO global test. To assess whether the overall causal effect was overly weighted toward individual SNPs, we ran leave‐one‐out sensitivity analyses. Scatterplots were generated to assess the robustness of the results. All analyses were conducted in R 4.3.2. The TwoSampleMR (version 0.6.8) and MR‐PRESSO (version 1.0) packages were used for most of the data analysis.

## 3 Results

### 3.1 Observational Study Findings

#### 3.1.1 The Baseline Characteristics of the Study Population

A total of 2578 older adults were included in the study. Table 1 gives the baseline demographic and clinical features of the participants (male: 45.77%; female: 54.23%). Non‐Hispanic whites were the largest group, accounting for more than 80% of the total sample.

**TABLE 1:** Basic information on the study population.

	Total (*N* = 2578)	Inactive (*N* = 1464)	Insufficiently active (*N* = 405)	Weekend warrior (*N* = 71)	Regularly active (*N* = 638)	*p*‐value
**Continuous variables (M ± SE)**						
CERAD (score)	0.25 (0.05)	0.15 (0.06)	0.37 (0.06)	0.18 (0.12)	0.38 (0.06)	< 0.001
AFT (score)	0.36 (0.04)	0.16 (0.03)	0.39 (0.07)	0.63 (0.20)	0.69 (0.06)	< 0.001
DSST (score)	0.40 (0.03)	0.21 (0.04)	0.50 (0.06)	0.63 (0.12)	0.69 (0.05)	< 0.001
Cognitive function (*Z* score)	0.43 (0.04)	0.24 (0.04)	0.53 (0.06)	0.60 (0.15)	0.73 (0.06)	< 0.001
**Categorical scalar *n* (%)**						
**Age**						0.023
60–69	1412 (56.82)	768 (53.64)	234 (57.75)	44 (59.61)	366 (62.13)	
70–79	761 (29.31)	443 (30.14)	120 (30.15)	21 (33.12)	177 (26.85)	
≥ 80	405 (13.87)	253 (16.22)	51 (12.10)	6 (7.28)	95 (11.02)	
**Sex**						0.042
Male	1249 (45.77)	687 (44.16)	184 (41.79)	42 (57.26)	336 (49.75)	
Female	1329 (54.23)	777 (55.84)	221 (58.21)	29 (42.74)	302 (50.25)	
**Race**						0.049
Non‐Hispanic Black	219 (3.23)	125 (3.43)	36 (3.32)	5 (2.22)	53 (2.91)	
Non‐Hispanic White	1264 (80.29)	723 (79.62)	176 (76.26)	37 (84.16)	328 (83.30)	
Mexican American	606 (8.12)	357 (8.83)	100 (9.31)	14 (5.29)	135 (6.40)	
Others	489 (8.37)	259 (8.12)	93 (11.12)	15 (8.33)	122 (7.39)	
**Marital status**						0.009
Married or living with a partner	1485 (64.80)	816 (62.80)	232 (62.37)	50 (76.09)	387 (68.73)	
Single (widowed/divorced/separated)	950 (30.98)	560 (32.53)	158 (35.51)	14 (15.72)	218 (27.23)	
Never married	143 (4.22)	88 (4.67)	15 (2.12)	7 (8.19)	33 (4.04)	
**Education**						< 0.001
High school grad or equivalent	620 (15.26)	437 (20.76)	78 (10.59)	10 (8.27)	95 (7.92)	
Less than high school	601 (21.50)	370 (23.86)	97 (23.58)	10 (12.06)	124 (16.88)	
Some college or above	1357 (63.25)	657 (55.39)	230 (65.83)	51 (79.67)	419 (75.20)	
**BMI**						
Underweight (< 18.5)	36 (1.28)	21 (1.25)	2 (1.07)	2 (2.16)	11 (1.36)	< 0.001
Normal (≥ 18.5 and ≤ 24.9)	649 (24.53)	329 (20.50)	101 (24.01)	19 (20.77)	200 (32.99)	
Overweight (> 24.9 and < 30)	908 (36.25)	506 (34.55)	135 (33.58)	22 (36.97)	245 (40.87)	
Obese (≥ 30)	985 (37.94)	608 (43.70)	167 (41.33)	28 (40.10)	182 (24.78)	
**PIR**						< 0.001
Low income (< 1.30)	761 (17.45)	516 (22.48)	93 (14.23)	13 (10.52)	139 (10.23)	
Middle income (1.30–3.49)	996 (38.52)	571 (40.46)	171 (38.10)	31 (47.34)	223 (34.03)	
High income (≥ 3.50)	821 (44.03)	377 (37.06)	141 (47.67)	27 (42.15)	276 (55.73)	
**Smoking status**						< 0.001
Nonsmoker	1270 (49.43)	688 (47.32)	208 (49.94)	31 (40.78)	343 (54.18)	
Former smoker	987 (39.52)	550 (37.62)	160 (41.73)	34 (53.94)	243 (40.41)	
Current smoker	321 (11.05)	226 (15.06)	37 (8.33)	6 (5.28)	52 (5.41)	
**Drinking status**						0.169
Mild	1812 (73.27)	997 (71.46)	290 (72.44)	59 (85.20)	466 (75.89)	
Moderate	586 (21.96)	352 (22.82)	92 (23.70)	7 (9.51)	135 (20.77)	
Heavy	180 (4.76)	115 (5.72)	23 (3.86)	5 (5.30)	37 (3.34)	
**Hypertension**						< 0.001
No	739 (32.55)	370 (27.35)	126 (36.49)	26 (30.74)	217 (40.68)	
Yes	1839 (67.45)	1094 (72.65)	279 (63.51)	45 (69.26)	421 (59.32)	
**Diabetes**						< 0.001
No	1687 (71.87)	915 (67.51)	262 (71.49)	49 (77.56)	461 (79.85)	
Yes	891 (28.13)	549 (32.49)	143 (28.51)	22 (22.44)	177 (20.15)	
**Depression**						< 0.001
No	2344 (92.70)	1294 (90.07)	368 (92.55)	69 (99.45)	613 (97.11)	
Yes	234 (7.30)	170 (9.93)	37 (7.45)	2 (0.55)	25 (2.89)	
**Sleep disorder**						0.003
No	2164 (85.41)	1191 (82.52)	343 (86.51)	59 (87.52)	571 (90.15)	
Yes	414 (14.59)	273 (17.48)	62 (13.49)	12 (12.48)	67 (9.85)	
**Stroke**						0.015
No	2402 (93.93)	1344 (92.50)	388 (96.89)	66 (96.56)	604 (94.79)	
Yes	176 (6.07)	120 (7.50)	17 (3.11)	5 (3.44)	34 (5.21)	

Abbreviations: AFT, animal fluency test; BMI, body mass index; CERAD, consortium to establish a registry for Alzheimer's disease word learning test; DSST, digit symbol substitution test; PIR, poverty income ratio.

Significant differences in PA pattern (*p* < 0.05) for gender, age, ethnicity, education, marital status, household income, smoking habit, and BMI were accounted for. These lifestyle differences significantly impact health and can influence brain function and overall well‐being. Further analysis revealed that compared to the completely inactive group, the CERAD, AFT, and DSST scores were progressively higher in the insufficiently active, WW, and RA groups.

Demographically, most “WW” patterns were among people aged 60–69, men, and those with higher education. Although they still engage in some physical exercise, their BMI is usually somewhat higher, suggesting a risk of obesity. But the “RA” pattern is most often seen in married individuals with an income/personal over 3.5 and nonsmokers. Regarding health outcomes, the “WW” group had relatively low self‐reported depressive symptoms, sleep problems, and stroke incidence.

#### 3.1.2 The Relationship Between Various PA Patterns and Specific Cognitive Domains

Results of regressions between different PA patterns and multiple CF tests are presented in Table 2. In unadjusted Model 1, compared to the inactive group, the insufficient group (*β* = 0.21; 95% CI: 0.06, 0.37; *p* = 0.012) and the RA group (*β* = 0.23; 95% CI: 0.13, 0.33; *p* < 0.001) had significantly higher CERAD test scores. In Model 2, after controlling for age, sex, and ethnicity, the insufficient group (*β* = 0.18; 95% CI: 0.04, 0.32; *p* = 0.017) and the RA group (*β* = 0.17; 95% CI: 0.08, 0.27; *p* = 0.002) remained significant. In the fully adjusted Model 3, the association between insufficient PA and CERAD scores was no longer significant (*β* = 0.10; 95% CI: −0.03, 0.24; *p* = 0.247) compared to Model 2, but the RA group was still significantly associated (*β* = 0.07; 95% CI: 0.02, 0.16; *p* = 0.018). The WW group was not associated with CERAD scores in any of the models.

**TABLE 2:** Association between physical activity patterns and domain‐specific cognitive function.

Cognitive function tests	Model 1		Model 2		Model 3	
	*β* (95% CI)	*p*‐value	*β* (95% CI)	*p*‐value	*β* (95% CI)	*p*‐value
**CERAD**						
Inactive	Reference		Reference		Reference	
Insufficiently active	0.21 (0.06, 0.37)	0.012	0.18 (0.04, 0.32)	0.017	0.10 (−0.03, 0.24)	0.247
Weekend warrior	0.03 (−0.22, 0.28)	0.839	−0.02 (−0.24, 0.20)	0.884	−0.12 (−0.33, 0.08)	0.290
Regularly active	0.23 (0.13, 0.33)	< 0.001	0.17 (0.08, 0.27)	0.002	0.07 (0.02, 0.16)	0.018
	*P* for trend	< 0.001	*P* for trend	< 0.001	*P* for trend	< 0.001
**AFT**						
Inactive	Reference		Reference		Reference	
Insufficiently active	0.23 (0.09, 0.37)	0.002	0.22 (0.09, 0.35)	0.003	0.12 (0.00, 0.25)	0.187
Weekend warrior	0.47 (0.08, 0.85)	0.024	0.37 (0.01, 0.72)	0.056	0.24 (0.11, 0.60)	0.238
Regularly active	0.53 (0.41, 0.66)	< 0.001	0.45 (0.33, 0.56)	< 0.001	0.31 (0.21, 0.41)	0.002
	*P* for trend	< 0.001	*P* for trend	< 0.001	*P* for trend	< 0.001
**DSST**						
Inactive	Reference		Reference		Reference	
Insufficiently active	0.29 (0.15, 0.42)	< 0.001	0.26 (0.14, 0.37)	< 0.001	0.12 (0.02, 0.21)	0.057
Weekend warrior	0.42 (0.19, 0.64)	0.001	0.33 (0.14, 0.52)	0.043	0.15 (0.03, 0.33)	0.155
Regularly active	0.48 (0.34, 0.62)	< 0.001	0.39 (0.26, 0.52)	< 0.001	0.19 (0.09, 0.30)	0.017
	*P* for trend	< 0.001	*P* for trend	< 0.001	*P* for trend	< 0.001
**Cognitive function (*Z*)**						
Inactive	Reference		Reference		Reference	
Insufficiently active	0.29 (0.16, 0.42)	< 0.001	0.26 (0.15, 0.37)	< 0.001	0.14 (0.03, 0.24)	0.053
Weekend warrior	0.36 (0.07, 0.65)	0.021	0.27 (0.03, 0.51)	0.036	0.11 (0.03, 0.34)	0.405
Regularly active	0.49 (0.38, 0.61)	< 0.001	0.40 (0.29, 0.51)	< 0.001	0.23 (0.14, 0.32)	0.004
	*P* for trend	< 0.001	*P* for trend	< 0.001	*P* for trend	0.001

*Note*: Model 1 was unadjusted model. Model 2 was adjusted for age, sex, and race. Model 3 was adjusted for age, sex, race, BMI, education level, marital status, PIR, smoking status, alcohol consumption, diabetes, hypertension, depressive symptoms, sleep disorders, and history of stroke.

Abbreviations: AFT, animal fluency test; CERAD, consortium to establish a registry for Alzheimer's disease word learning test; DSST, digit symbol substitution test.

In the AFT test, Model 1 showed that insufficient (*β* = 0.23; 95% CI: 0.09, 0.37; *p* = 0.002), WW (*β* = 0.47; 95% CI: 0.08, 0.85; *p* = 0.024), and RA (*β* = 0.53; 95% CI: 0.41, 0.66; *p* < 0.001) had significantly higher scores than the inactive group. Adjusted for demographic variables in Model 2, the insufficient group (*β* = 0.22; 95% CI: 0.09, 0.35; *p* = 0.003) and the RA group (*β* = 0.45; 95% CI: 0.33, 0.56; *p* < 0.001) remained significant, but the WW group was not. After additional adjustment for all confounders in Model 3, only the RA group retained an independent association with AFT scores (*β* = 0.31; 95% CI: 0.21, 0.41; *p* = 0.002).

As for DSST and composite CF (*Z*‐score), Model 1 and Model 2 showed that the insufficient, WW, and RA groups had higher scores than the inactive group (*p* < 0.05). In Model 3, only the RA group showed a significant positive correlation with CF scores. These findings imply that being physically active at least three times a week has cognitive‐protective benefits for people aged 60 and older. In contrast, the WW activity pattern has limited effectiveness at improving cognitive performance.

#### 3.1.3 PA Intensity and Specific Cognitive Domains

Table 3 presents the regression analysis results for different levels of PA intensity and cognitive domains. Compared with the non‐exercising group, MPA did not show a significant association with CERAD scores in Models 1, 2, and 3, whereas VPA showed a significant positive association across all models (*p* < 0.05). AFT test results showed that MPA was significantly related to Model 1 and Model 2. Similarly, VPA was significantly related to Model 1 and Model 2. In Model 3, which accounted for all confounders, only VPA remained significant, whereas the association for MPA became nonsignificant. For DSST and composite cognitive *Z*‐score, Models 1–3 revealed that MPA and VPA were significantly associated with each other in the inactive group, regardless of covariate adjustment (*p* < 0.05). Trend analysis showed that the CF score increased progressively with increasing PA intensity. Trend analysis showed a strong positive relationship between PA intensity and cognitive performance (trend *p* < 0.001).

**TABLE 3:** Association between different physical activity intensities and domain‐specific cognitive function.

Cognitive function tests	Model 1		Model 2		Model 3	
	*β* (95% CI)	*p*‐value	*β* (95% CI)	*p*‐value	*β* (95% CI)	*p*‐value
**CERAD**						
Inactive	Reference		Reference		Reference	
MPA	0.00 (0.00, 0.00)	0.875	0.00 (0.00, 0.00)	0.675	0.00 (0.00, 0.00)	0.169
VPA	0.01 (0.01, 0.01)	0.001	0.01 (0.01, 0.01)	0.005	0.00 (0.00, 0.00)	0.037
	*P* for trend	< 0.001	*P* for trend	< 0.001	*P* for trend	< 0.001
**AFT**						
Inactive	Reference		Reference		Reference	
MPA	0.00 (0.00, 0.01)	0.001	0.00 (0.00, 0.01)	0.005	0.00 (0.00, 0.00)	0.055
VPA	0.01 (0.01, 0.01)	< 0.001	0.01 (0.01, 0.01)	< 0.001	0.01 (0.01, 0.01)	0.002
	*P* for trend	< 0.001	*P* for trend	< 0.001	*P* for trend	< 0.001
**DSST**						
Inactive	Reference		Reference		Reference	
MPA	0.01 (0.01, 0.01)	0.024	0.01 (0.01, 0.01)	0.028	0.00 (0.00, 0.00)	0.045
VPA	0.01 (0.01, 0.01)	< 0.001	0.01 (0.01, 0.01)	< 0.001	0.01 (0.01, 0.01)	0.003
	*P* for trend	< 0.001	*P* for trend	< 0.001	*P* for trend	< 0.001
**Cognitive function (*Z*)**						
Inactive	Reference		Reference		Reference	
MPA	0.01 (0.01, 0.01)	0.022	0.01 (0.01, 0.01)	0.029	0.00 (0.00, 0.00)	0.043
VPA	0.01 (0.01, 0.01)	< 0.001	0.01 (0.01, 0.01)	< 0.001	0.01 (0.01, 0.01)	0.001
	*P* for trend	< 0.001	*P* for trend	< 0.001	*P* for trend	< 0.001

*Note*: Model 1 was unadjusted model. Model 2 was adjusted for age, sex, and race. Model 3 was adjusted for age, sex, race, BMI, education level, marital status, PIR, smoking status, alcohol consumption, diabetes, hypertension, depressive symptoms, sleep disorders, and history of stroke.

Abbreviations: AFT, animal fluency test; CERAD, consortium to establish a registry for Alzheimer's disease word learning test; DSST, digit symbol substitution test; MPA, moderate‐intensity physical activity; PA, physical activity; VPA, vigorous‐intensity physical activity.

#### 3.1.4 PA's Association With Certain Cognitive Domains and Threshold Effect Analysis

**FIGURE 2:**
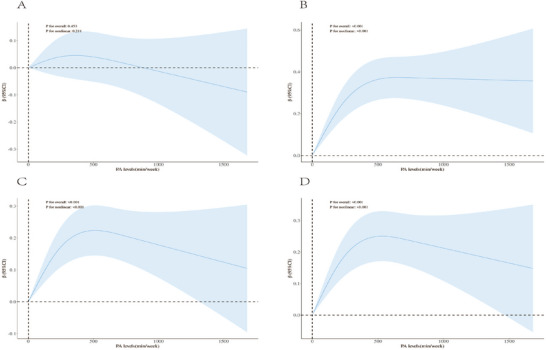
Inverted U‐shaped relationship between physical activity and domain‐specific cognitive function scores.

Restricted cubic spline curve analysis (Figure 2) and threshold effect analysis (Table 4) both indicated a nonlinear relationship between PA and AFT, DSST and composite cognitive *Z*‐scores, with an overall inverted U‐shaped pattern. As we look at higher PA levels, these cognitive measures tend to go up, but start going down after a certain level is reached, an inflection point. The inflection points were AFT at 650 min/week, DSST at 535 min/week, and the composite cognitive *Z*‐score at 550 min/week.

**TABLE 4:** Threshold effect analysis of different physical activity intensities on domain‐specific cognitive function.

	CERAD	AFT	DSST	Cognitive function (*Z*)
	*β* (95% CI) *p*‐value			
One‐line linear regression model	0.01 (−0.00, 0.02) 0.661	0.00 (0.00, 0.01) 0.001	0.01 (0.00, 0.02) < 0.001	0.01 (0.00, 0.02) < 0.001
Two‐piecewise linear regression model				
Inflection point (K)	420	650	535	550
< K (minutes/week)	0.01 (−0.00, 0.02) 0.218	0.00 (0.00, 0.01) 0.006	0.01 (0.01, 0.02) 0.012	0.01 (0.00, 0.02) < 0.001
≥ K (minutes/week)	−0.00 (−0.01, 0.01) 0.392	−0.00 (−0.00, 0.00) 0.859	−0.00 (−0.00, 0.00) 0.205	−0.01 (−0.02, 0.00) 0.235
Log‐likelihood ratio	0.207	< 0.001	< 0.001	< 0.001

Abbreviations: AFT, animal fluency test; CERAD, consortium to establish a registry for Alzheimer's disease word learning test; DSST, digit symbol substitution test.

Below the inflection point, each minute of PA added was associated with a 0.00‐point increase in AFT scores and 0.01‐point increase in both DSST and composite cognitive *Z*‐scores. Past the inflection points, the relationship between PA and AFT or DSST became nonsignificant, with each minute of PA decreasing the composite cognitive *Z*‐score by 0.01 points. There is no significant linear relationship between PA and CERAD score.

#### 3.1.5 Subgroup Analysis

Table 5 shows the association between PA and cognitive performance across subgroups. With CERAD and AFT used as the outcomes, the positive link between PA and cognitive performance stayed constant among subgroups based on age, sex, household income, smoking, hypertension, diabetes, depression, sleep disorders, and stroke. However, for interaction with DSST (*p* = 0.024) or the composite cognitive score (*p* = 0.028) as outcomes, depression moderated the association between PA and CF, but was stable across all other subgroups.

**TABLE 5:** Subgroup analysis of physical activity and domain‐specific cognitive function.

	CERAD	AFT	DSST	Cognitive function (*Z*)
Variables	*β* (95% CI)	*β* (95% CI)	*β* (95% CI)	*β* (95% CI)
**Age**				
60–69	−0.00 (−0.00, 0.00)	0.00 (0.00, 0.01)	0.00 (−0.00, 0.00)	0.00 (0.00, 0.01)
70–79	−0.00 (−0.00, 0.00)	0.00 (0.00, 0.01)	0.00 (0.00, 0.01)	0.00 (0.00, 0.01)
≥ 80	0.00 (−0.00, 0.00)	0.00 (0.00, 0.01)	0.00 (0.00, 0.01)	0.00 (0.00, 0.00)
*P* for interaction	0.862	0.607	0.234	0.522
**Sex**				
Male	−0.00 (−0.00, 0.00)	0.00 (0.00, 0.01)	0.00 (0.00, 0.00)	0.00 (0.00, 0.00)
Female	0.00 (−0.00, 0.00)	0.00 (0.00, 0.01)	0.00 (0.00, 0.00)	0.00 (0.00, 0.01)
*P* for interaction	0.494	0.778	0.895	0.607
**PIR**				
Low income (< 1.30)	−0.00 (−0.00, 0.00)	0.00 (−0.00,0.00)	0.00 (0.00, 0.01)	0.00 (−0.00, 0.00)
Middle income (1.30–3.49)	0.00 (−0.00, 0.00)	0.00 (0.00, 0.01)	0.00 (−0.00, 0.00)	0.00 (0.00, 0.01)
High income (≥ 3.50)	−0.00 (−0.00, 0.00)	0.00 (0.00, 0.01)	0.00 (−0.00, 0.00)	0.00 (0.00, 0.01)
*P* for interaction	0.434	0.089	0.392	0.720
**Smoking status**				
Nonsmoker	−0.00 (−0.00, 0.00)	0.00 (0.00, 0.01)	0.00 (0.00, 0.01)	0.00 (0.00, 0.01)
Former smoker	0.00 (−0.00, 0.00)	0.00 (0.00, 0.00)	0.00 (−0.00, 0.00)	0.00 (0.00, 0.00)
Current smoker	0.00 (−0.00, 0.00)	0.00 (0.00, 0.01)	0.00 (−0.00, 0.00)	0.00 (0.00, 0.00)
*P* for interaction	0.949	0.323	0.430	0.429
**Hypertension**				
No	−0.00 (−0.00, 0.00)	0.00 (0.00, 0.01)	0.00 (−0.00, 0.00)	0.00 (0.00, 0.00)
Yes	0.00 (−0.00, 0.00)	0.00 (0.00, 0.01)	0.00 (0.00, 0.01)	0.00 (0.00, 0.01)
*P* for interaction	0.937	0.128	0.255	0.922
**Diabetes**				
No	−0.00 (−0.00, 0.00)	0.00 (0.00, 0.01)	0.00 (0.00, 0.01)	0.00 (0.00, 0.01)
Yes	0.00 (−0.00, 0.00)	0.00 (0.00, 0.01)	0.00 (−0.00, 0.00)	0.00 (0.00, 0.01)
*P* for interaction	0.738	0.816	0.396	0.618
**Depression**				
No	−0.00 (−0.00, 0.00)	0.00 (0.00, 0.01)	0.00 (0.00, 0.01)	0.00 (0.00, 0.01)
Yes	0.00 (−0.00, 0.00)	0.00 (0.00, 0.01)	0.00 (0.00, 0.01)	0.00 (0.00, 0.01)
*P* for interaction	0.407	0.181	0.024	0.028
**Sleep disorder**				
No	−0.00 (−0.00, 0.00)	0.00 (0.00, 0.01)	0.00 (0.00, 0.01)	0.00 (0.00, 0.01)
Yes	0.00 (−0.00, 0.00)	0.00 (0.00, 0.00)	0.00 (−0.00, 0.00)	0.00 (0.00, 0.01)
*P* for interaction	0.673	0.958	0.563	0.522
**Stroke**				
No	−0.00 (−0.00, 0.00)	0.00 (0.00, 0.01)	0.00 (0.00, 0.01)	0.00 (0.00, 0.01)
Yes	0.00 (−0.00, 0.00)	0.00 (−0.00, 0.00)	0.00 (0.00, 0.01)	0.00 (0.00, 0.00)
*P* for interaction	0.837	0.577	0.140	0.466

*Note*: Model 3 was adjusted for age, sex, race, BMI, education level, marital status, PIR, smoking status, alcohol consumption, diabetes, hypertension, depressive symptoms, sleep disorders, and history of stroke.

Abbreviations: AFT, animal fluency test; CERAD, consortium to establish a registry for Alzheimer's disease word learning test; DSST, digit symbol substitution test; PIR, poverty income ratio.

### 3.2 MR Study Results

In this study, we use an MR approach to examine the causal effects of MPA and VPA on five CF indicators: memory performance, reaction time, correct matching count, fluid intelligence, and overall cognitive performance. MPA analysis used 17, 8, 13, 7, and 10 SNPs as IVs, respectively; and VPA analysis selected 11, 7, 10, 6, and 6 SNPs. All SNPs showed *F*‐stat > 10, indicating that the IVs were strongly associated with the exposure factors and that they controlled for weak IV bias (Supporting Information S2).

In analyses with MPA as the exposure factor, results differed by cognitive domain. For memory performance and reaction time, there were no significant causal associations for any of the methods: IVW, MR‐Egger, weighted median, and MR‐RAPS (all *p* > 0.05). Analyses with the correct matching count as the outcome show that all four methods yield consistent results showing a significant causal effect of MPA—IVW: OR = 0.710, 95% CI: 0.606, 0.832, *p* < 0.001; MR‐Egger: OR = 0.370, 95% CI: 0.165, 0.827, *p* = 0.033; weighted median method: OR = 0.844, 95% CI: 0.729, 0.977, *p* = 0.023; MR‐RAPS: OR = 0.687, 95% CI: 0.565, 0.836, *p* < 0.001. Fluid intelligence analysis, IVW (OR = 0.470; 95% CI: 0.346, 0.638; *p* < 0.001), weighted median method (OR = 0.528; 95% CI: 0.391, 0.715; *p* < 0.001), and MR‐RAPS (OR = 0.470; 95% CI: 0.331, 0.669; *p* < 0.001) indicate significant causal effect, while MR‐Egger does not show statistical significance. For the overall cognitive performance, IVW (OR = 0.707; 95% CI: 0.653, 0.764; *p* < 0.001), weighted median method (OR = 0.745; 95% CI: 0.627, 0.827; *p* < 0.001), and MR‐RAPS (OR = 0.706; 95% CI: 0.650, 0.767; *p* < 0.001) had significant effects but MR‐Egger didn't (Table 6).

**TABLE 6:** Results from the Mendelian randomization study of the association between physical activity and domain‐specific cognitive function.

Exposure	Outcome	SNPs	MR		
			Method	OR (95% CI)	*P*
MPA	Memory performance	17	IVW	0.933 (0.851, 1.022)	0.140
			Weighted median	0.925 (0.819, 1.045)	0.213
			MR‐Egger	1.005 (0.587, 1.721)	0.983
			MR‐RAPS	0.935 (0.849, 1.029)	0.173
MPA	Reaction time	8	IVW	1.016 (0.543, 1.900)	0.958
			Weighted median	1.042 (0.479, 2.268)	0.915
			MR‐Egger	0.732 (0.193, 1.435)	0.872
			MR‐RAPS	1.016 (0.522, 1.977)	0.961
MPA	Number of correct matches	13	IVW	0.710 (0.606, 0.832)	< 0.001
			Weighted median	0.844 (0.729, 0.977)	0.023
			MR‐Egger	0.370 (0.165, 0.827)	0.033
			MR‐RAPS	0.687 (0.565, 0.836)	< 0.001
MPA	Fluid intelligence	7	IVW	0.470 (0.346, 0.638)	< 0.001
			Weighted median	0.528 (0.391, 0.715)	< 0.001
			MR‐Egger	1.473 (0.650, 1.973)	0.162
			MR‐RAPS	0.470 (0.331, 0.669)	< 0.001
MPA	Cognitive performance	10	IVW	0.707 (0.653, 0.764)	< 0.001
			Weighted median	0.745 (0.627, 0.827)	< 0.001
			MR‐Egger	0.845 (0.424, 1.682)	0.644
			MR‐RAPS	0.706 (0.650, 0.767)	< 0.001
VPA	Memory performance	11	IVW	0.850 (0.726, 0.996)	0.044
			Weighted median	0.765 (0.664, 0.906)	0.001
			MR‐Egger	0.898 (0.240, 3.36)	0.877
			MR‐RAPS	0.830 (0.714, 0.965)	0.015
VPA	Reaction time	7	IVW	0.368 (0.164, 0.824)	0.015
			Weighted median	0.518 (0.180, 1.488)	0.222
			MR‐Egger	1.257 (0.441, 1.787)	0.559
			MR‐RAPS	0.388 (0.164, 0.918)	0.031
VPA	Number of correct matches	10	IVW	0.616 (0.542, 0.699)	< 0.001
			Weighted median	0.666 (0.573, 0.774)	< 0.001
			MR‐Egger	0.160 (0.072, 0.357)	< 0.001
			MR‐RAPS	0.604 (0.522, 0.699)	< 0.001
VPA	Fluid intelligence	6	IVW	0.660 (0.521, 0.835)	< 0.001
			Weighted median	0.622 (0.457, 0.848)	0.002
			MR‐Egger	1.480 (0.450, 2.936)	0.355
			MR‐RAPS	0.650 (0.503, 0.840)	< 0.001
VPA	Cognitive performance	6	IVW	0.768 (0.663, 0.889)	< 0.001
			Weighted median	0.792 (0.694, 0.903)	< 0.001
			MR‐Egger	0.571 (0.180, 1.805)	0.394
			MR‐RAPS	0.758 (0.648, 0.887)	< 0.001

Abbreviations: IVW, inverse‐variance weighted; MPA, moderate‐intensity physical activity; VPA, vigorous‐intensity physical activity.

Analyses with VPA as the exposure factor, memory performance showed significant effect for IVW (OR = 0.850; 95% CI: 0.726, 0.996; *p* = 0.044), weighted median method (OR = 0.765; 95% CI: 0.664, 0.906; *p* = 0.001) and MR‐RAPS (OR = 0.830; 95% CI: 0.714, 0.965; *p* = 0.015) were significant causal, but MR‐Egger did not. In the reaction time analysis, IVW (OR = 0.368; 95% CI: 0.164, 0.824; *p* = 0.015) and MR‐RAPS (OR = 0.388; 95% CI: 0.164, 0.918; *p* = 0.031) showed statistically significant results, whereas the weighted median and MR‐Egger methods did not. In the exact matching, IVW (OR = 0.616; 95% CI: 0.542, 0.699; *p* < 0.001), MR‐Egger (OR = 0.160; 95% CI: 0.072, 0.357; *p* < 0.001), weighted median (OR = 0.666; 95% CI: 0.573, 0.774; *p* < 0.001), and MR‐RAPS (OR = 0.604; 95% CI: 0.522, 0.699; *p* < 0.001) all show a causal effect. Analysis of fluid intelligence and overall cognitive performance produced causation signals from all methods, including IVW, weighted median, and MR‐RAPS, but not MR‐Egger (Table 6).

The reverse MR analysis showed no causal effect of VPA on CF indicators. In the analyses using memory performance as exposure and MPA as outcome, both the IVW (OR = 1.039; 95% CI: 1.003, 1.076; *p* = 0.032) and weighted median methods (OR = 1.057; 95% CI: 1.015, 1.076; *p* = 0.007) yielded results suggesting a possible causation, whereas the MR‐Egger and MR‐RAPS analyses did not show any significant results (Table S1). Sensitivity analysis indicated that Cochran's *Q* test detected heterogeneity for MPA regarding the number of correctly matched pairs (*p* < 0.001); thus, we corrected for it using a random‐effects IVW model. No significant heterogeneity was seen in the other analyses. The MR‐Egger intercept and the MR‐PRESSO global test did not indicate overall multiplicity (*p* > 0.05). Leave‐one‐out analysis did not show a major difference in results when removing any SNPs; scatterplot analysis also supported robustness (Tables S2 and S3) (Figures S1–S4).

## 4 Discussion

### 4.1 Summary of Associations and Causal Findings

This study combined cross‐sectional survey data with MR analyses to investigate the relationship between PA and specific domains of CF. In the cross‐sectional analysis, when PA was treated as a categorical variable, all PA patterns, including insufficient activity, the WW pattern, and RA, were associated with higher cognitive scores compared with the inactive group. However, only the RA pattern demonstrated consistent and significant positive associations across specific cognitive domains, whereas the insufficiently active and WW patterns did not show substantial or stable effects. When PA was analyzed as a continuous variable, VPA showed positive correlations with several indicators of CF. MPA did not exhibit strong causal relationships with memory‐related dimensions, but it was positively associated with AFT, DSST, and overall cognitive performance. In addition, this study identified an inverted U‐shaped relationship between MVPA and AFT, DSST, and overall cognitive performance scores. Further stratified analyses revealed no obvious interactions between PA and demographic factors except for depression, suggesting that the influence of PA on CF was generally similar across population subgroups. With regard to depression, the data indicated that the association between PA and CF, particularly in relation to DSST scores and overall CF, may be moderated by depressive status. In the MR analyses, no causal association was identified between MPA and memory performance. However, significant causal effects were observed between MPA and reaction time, correct matches, fluid intelligence, and overall cognitive level. VPA showed significant associations with memory performance, reaction time, correct matches, fluid intelligence, and overall CF. In the reverse‐causality analyses, only memory performance showed a reverse causal effect on MPA, whereas no significant reverse causal relationships were observed between PA and the other cognitive measures.

### 4.2 The Promotive Role of PA in CF Among Older Adults and Its Underlying Mechanisms

The findings of this study are generally consistent with previous literature, further confirming the role of PA in promoting CF in older adults and supporting the adoption of an active lifestyle as an important strategy for maintaining cognitive health (Feter et al. 2024; Gu et al. 2020; López‐Bueno et al. 2023). Although the optimal temporal distribution pattern of PA remains inconclusive, the present study suggests that, compared with physical inactivity, nearly all forms of PA are associated with improved performance in specific cognitive domains. The protective effects of PA on cognition may be mediated through multidimensional physiological mechanisms. At the level of brain structure and function, PA has been shown to induce structural remodeling, increase gray and white matter volume (Colcombe et al. 2006; Erickson et al. 2012; Miller et al. 2012), improve neuronal metabolic markers to preserve neural integrity (Erickson et al. 2014; Matura et al. 2017), and enhance cerebral blood flow by promoting angiogenesis (Bherer et al. 2013). Long‐term observations further indicate that these changes primarily occur in key brain regions involved in cognition, including the frontal lobe (Erickson et al. 2012), hippocampus and prefrontal cortex (Erickson et al. 2014), as well as the temporal lobe (Colcombe et al. 2006). At the systemic physiological level, PA contributes to reduced cardiovascular disease risk and suppression of inflammatory responses, both of which are important risk factors for cognitive decline and neurodegenerative disorders (Hamer and Chida 2009). In addition, PA may upregulate the expression of neurotrophic factors such as vascular endothelial growth factor (VEGF), thereby enhancing brain function through the stimulation of neurogenesis and angiogenesis (Vital et al. 2014). Regarding activity patterns, this study found that short‐term but intensive PA, represented by the WW pattern, was associated with improvements in reaction speed, executive function, and overall cognitive performance. However, the strength of these associations weakened after adjustment for multiple confounding variables. This finding differs from previous studies reporting robust benefits of the WW pattern and may be attributable to heterogeneity in study populations. For example, earlier studies mainly focused on older women, who may experience accelerated cognitive decline after menopause due to declining estrogen levels and may therefore be more responsive to short‐term PA interventions (Wu et al. 2024b). Overall, regardless of the PA pattern adopted, as long as it is appropriate for the target population and can generate cognitive benefits, even low‐dose PA still has substantial value for health promotion.

### 4.3 The Relative Advantages of VPA in Promoting Cognitive Health and Its Dose‐Threshold Effects

Consistent findings from both the cross‐sectional analyses and MR results suggest that VPA may have a greater advantage than MPA in promoting cognitive health, highlighting the importance of placing greater emphasis on VPA in exercise intervention recommendations for older adults. Mechanistically, VPA may improve CF through several synergistic pathways. On the one hand, VPA has been shown to upregulate brain‐derived neurotrophic factor (BDNF), thereby enhancing neuronal survival, synaptic plasticity, and neurogenesis, which in turn may optimize learning, memory, and executive function and delay the pathological progression of dementia‐related disorders (Cotman and Berchtold 2002; Mata et al. 2010; Sanders et al. 2020). On the other hand, VPA may improve cerebral perfusion, increase oxygen delivery to brain tissue, accelerate the clearance of neurotoxic metabolites such as β‐amyloid, and attenuate neuroinflammatory processes, thereby contributing to the maintenance of structural brain integrity and the prevention of neurodegenerative diseases (Augusto‐Oliveira et al. 2023; Azevedo et al. 2023; Nguyen et al. 2023; Ren et al. 2024). Notably, this study further identified significant threshold effects between MVPA and AFT, DSST, as well as overall CF. Specifically, the data indicated that when weekly MVPA duration exceeded 650, 535, and 550 min, respectively, the marginal cognitive benefits gradually plateaued or even declined. This finding provides a novel perspective on the current literature and suggests that recommendations for PA in older adults should take into account the nonlinear relationship between PA volume and cognitive benefit. Unlike previous studies that mainly emphasized a linear positive relationship between exercise duration and CF, the threshold effect identified here offers empirical support for the development of more precise, feasible, and individualized cognitive‐enhancement programs for older adults, with clearer practical implications.

### 4.4 The Moderating Role of Depression in the Association Between PA and CF and the Potential Bidirectional Relationship With Memory Function

Subgroup analyses revealed that depression significantly moderated the associations between PA and DSST scores, as well as overall CF. Previous studies have suggested that individuals with depressive symptoms may experience more pronounced cognitive benefits from engaging in PA (Wu et al. 2024a). PA not only alleviates the severity of depressive symptoms but may also reduce the risk of depression onset and recurrence (Liang et al. 2023). These mental health benefits are particularly important given that lifestyle‐related risk factors, such as prolonged sedentary behavior, have been independently associated with depressive symptoms (Guo et al. 2024b). In addition, environmental stressors, including recent tobacco smoking and exposure to secondhand smoke in public settings, have also been identified as important correlates of depression among adults, which further underscores the value of PA as a protective behavioral intervention (Mo et al. 2024).

Potential mechanisms may involve the regulation of mood‐related monoamine neurotransmitter activity by PA, thereby influencing cognitive processes (Xu et al. 2024). At the same time, monoaminergic signaling pathways may regulate BDNF gene expression and promote hippocampal BDNF expression, which may represent a key mechanism through which PA improves depression and related cognitive impairments (Castrén and Monteggia 2021). Reverse MR analyses suggested that memory performance may exert a causal effect on MPA. In contrast, forward MR analyses did not identify a causal influence of MPA on memory performance, but only observed an association between VPA and memory performance. Previous longitudinal studies have shown that PA levels are associated with working memory, as assessed by digit span tests (Weuve et al. 2004), whereas other studies failed to identify significant associations between PA and verbal memory (Brewster et al. 2014; Sattler et al. 2011), suggesting that the effects of PA on different memory domains may be selective rather than uniform.

As a core component of executive function, working memory may benefit from PA. At the same time, individuals with better memory capacity may be more likely to adhere to exercise regimens, which is associated with stronger self‐management ability and behavioral consistency (Raffin et al. 2023). Furthermore, good physical health provides the foundation for older adults to participate in PA, enabling them to manage their health more effectively and potentially slow the aging process through regular exercise (Junger and van Kampen 2010). Taken together, these findings suggest that the relationship among PA, depression, and memory‐related CF may be more complex than a simple one‐way association, and may instead involve interrelated behavioral, psychological, and neurobiological pathways.

### 4.5 Strengths and Limitations

A key strength of this study is the combination of NHANES 2011–2014 cross‐sectional data with MR analyses to investigate the relationship between PA and domain‐specific CF from both observational and causal perspectives. This integrated design provides a more comprehensive understanding of the PA–cognition association while reducing reliance on large‐scale randomized controlled trials or long‐term cohort studies.

Several limitations should also be acknowledged. First, the NHANES sample was drawn from a multiethnic older US population, whereas the genetic datasets used for MR were primarily based on European populations. We did not perform ancestry‐specific validation; therefore, the generalizability of the MR findings to non‐European populations remains uncertain. Second, the WW group was relatively small (*N* = 71, 2.7% of the total sample), which may have reduced the stability of the corresponding estimates. Thus, findings related to this subgroup should be interpreted cautiously. Third, the NHANES data were collected between 2011 and 2014, and lifestyle behaviors among older adults may have changed over the past decade, which may limit the direct applicability of these findings to the current elderly population. Fourth, PA was assessed by self‐report, which may have introduced recall bias and measurement error, despite the use of a validated questionnaire. Fifth, although NHANES included several cognitive tests, not all cognitive domains were covered. Finally, residual confounding from unmeasured factors, such as diet, social engagement, or other lifestyle behaviors, cannot be fully excluded. Future studies based on larger, more recent, and more diverse populations are needed to validate and extend these findings.

## 5 Conclusion

In conclusion, our study shows that any type of PA is associated with improved cognitive performance in a specific domain compared with inactivity. Among various activity patterns, the RA pattern demonstrates more stable and persistent associations with cognitive benefits in older adults, suggesting its particular suitability for this population. Both cross‐sectional and MR analyses indicate that VPA has significant positive effects on multiple cognitive measures, providing support for recommending higher‐intensity exercise in health guidance for older adults.

## Author Contributions

**Xingxiao Yin**: conceptualization, methodology, writing – original draft, writing – review and editing. **Hao Peng**: conceptualization, software, data curation. **Yanqi Li**: conceptualization, validation, investigation. **Yanping Song**: conceptualization, formal analysis, visualization. **Longjiang Chen**: conceptualization, formal analysis, visualization. **Na Yao**: conceptualization, data curation. **Pengcheng Li**: conceptualization, software, validation. **Zhijuan He**: conceptualization, software, validation. **Hongbo Chen**: conceptualization, validation, visualization. **Li Huang**: visualization, methodology. **Zhen Shen**: project administration, resources, writing – review and editing, funding acquisition. **Qigang Chen**: project administration, resources, writing – review and editing, funding acquisition.

## Funding

This research was supported by the National Natural Science Foundation of China (82360943).

## Ethics Statement

The data analyzed in this study were obtained from the National Health and Nutrition Examination Survey (NHANES) public database. The NHANES protocol was approved by the National Center for Health Statistics (NCHS) Ethics Review Board, and all participants provided written informed consent. As this study is a secondary analysis of a de‐identified public database, further ethical approval was not required.

## Conflicts of Interest

The authors declare no conflicts of interest.

## Supporting information




**Supplementary Information**: brb371423‐sup‐0001‐SuppMat.docx

## Data Availability

The datasets analyzed during the current study are available in the National Health and Nutrition Examination Survey (NHANES) repository, which is publicly accessible at: https://wwwn.cdc.gov/nchs/nhanes/.
