# Supplementary material for: The Association Between Physical Activity and Domain‐Specific Cognitive Function in the Elderly: A Cross‐Sectional Study and Genetic Analysis
Source: Brain Behav. 2026 May 5;16(5):e71423. doi: 10.1002/brb3.71423 (PMC13144551; doi:10.1002/brb3.71423)
Supplement: Supplementary file 1 — Supplementary Information: brb371423‐sup‐0001‐SuppMat.docx [file BRB3-16-e71423-s001.docx]

**Table S1: Results from the Mendelian randomization study of the association between domain-specific cognitive function and physical activity.**

| **Exposure** | **Outcome** | **SNPs** | **MR** | | |
| --- | --- | --- | --- | --- | --- |
|  |  |  | **Method** | **OR (95%CI)** | **P** |
| Memory Performance | MPA | 11 | IVW | 1.039(1.003,1.076) | 0.032 |
|  |  |  | Weighted median | 1.057(1.015,1.076) | 0.007 |
|  |  |  | MR-Egger | 1.054(0.965,1.154) | 0.268 |
|  |  |  | MR-RAPS | 1.042(0.977,1.111) | 0.207 |
| Reaction time | MPA | 7 | IVW | 0.997(0.990,1.005) | 0.585 |
|  |  |  | Weighted median | 0.998(0.989,1.007) | 0.707 |
|  |  |  | MR-Egger | 0.997(0.981,1.013) | 0.792 |
|  |  |  | MR-RAPS | 0.977(0.989,1.005) | 0.604 |
| Number of correct matches | MPA | 6 | IVW | 0.978(0.916,1.046) | 0.529 |
|  |  |  | Weighted median | 0.986(0.927,1.049) | 0.670 |
|  |  |  | MR-Egger | 0.930(0.752,1.149) | 0.538 |
|  |  |  | MR-RAPS | 0.979(0.919,1.043) | 0.516 |
| Fluid Intelligence | MPA | 52 | IVW | 0.978(0.913,1.043) | 0.562 |
|  |  |  | Weighted median | 0.984(0.922,1.057) | 0.691 |
|  |  |  | MR-Egger | 0.933(0.794,1.210） | 0.729 |
|  |  |  | MR-RAPS | 0.974(0.912,1.054) | 0.573 |
| Cognitive performance | MPA | 93 | IVW | 0.942(0.917,1.057) | 0.543 |
|  |  |  | Weighted median | 0.952(0.938,1.042) | 0.633 |
|  |  |  | MR-Egger | 0.929(0.849,1.139) | 0.539 |
|  |  |  | MR-RAPS | 0.939(0.911,1.043) | 0.529 |
| Memory Performance | VPA | 12 | IVW | 0.955(0.851,1.071) | 0.435 |
|  |  |  | Weighted median | 0.966(0.848,1.099) | 0.607 |
|  |  |  | MR-Egger | 1.098(0.860,1.400) | 0.468 |
|  |  |  | MR-RAPS | 0.980(0.856,1,123) | 0.781 |
| Reaction time | VPA | 7 | IVW | 1.006(0.974,1.038) | 0.702 |
|  |  |  | Weighted median | 0.988(0.957,1.020) | 0.481 |
|  |  |  | MR-Egger | 0.973(0.909,1.041) | 0.471 |
|  |  |  | MR-RAPS | 0.997(0.971,1.023) | 0.823 |
| Number of correct matches | VPA | 6 | IVW | 0.977(0.808,1.182) | 0.816 |
|  |  |  | Weighted median | 1.096(0.892,1.348) | 0.380 |
|  |  |  | MR-Egger | 0.808(0.439,1.488) | 0.532 |
|  |  |  | MR-RAPS | 0.970(0.800,1.177) | 0.763 |
| Fluid Intelligence | VPA | 51 | IVW | 0.977(0.917,1.047) | 0.639 |
|  |  |  | Weighted median | 0.982(0.923,1.059) | 0.732 |
|  |  |  | MR-Egger | 1.032(0.721,1.523) | 0.629 |
|  |  |  | MR-RAPS | 0.969(0.911,1.043) | 0.594 |
| Cognitive performance | VPA | 95 | IVW | 0.978(0.913,1.433) | 0.521 |
|  |  |  | Weighted median | 0.989(0.919,1.353) | 0.630 |
|  |  |  | MR-Egger | 1.032(0.793,1.521) | 0.599 |
|  |  |  | MR-RAPS | 0.970(0.903,1.342) | 0.639 |

**Abbreviations:** MPA: Moderate-Intensity Physical Activity

VPA: Vigorous-Intensity Physical Activity

IVW: Inverse-variance weighted

**Table S2: Results from the Mendelian randomization sensitivity analysis of the association between physical activity and domain-specific cognitive function.**

| **Exposure** | **Outcome** | **Heterogeneity**  **(Cochran’s Q test)** | | | | **Pleiotropy** | | |
| --- | --- | --- | --- | --- | --- | --- | --- | --- |
|  |  | **MR-egger** | | **IVW** | | **MR-egger** | | **MR-PRESSO** |
|  |  | **Q** | **P** | **Q** | **P** | **Intercept** | **P** | **P** |
| MPA | Memory Performance | 16.095 | 0.307 | 16.183 | 0.369 | -0.002 | 0.786 | 0.375 |
| MPA | Reaction time | 2.461 | 0.870 | 2.497 | 0.927 | 0.010 | 0.861 | 0.924 |
| MPA | Number of correct matches | 42.729 | <0.001 | 52.874 | <0.001 | 0.002 | 0.134 | 0.169 |
| MPA | Fluid Intelligence | 13.517 | 0.184 | 14.828 | 0.211 | -0.090 | 0.077 | 0.063 |
| MPA | Cognitive performance | 14.854 | 0.0629 | 15.33 | 0.082 | -0.005 | 0.623 | 0.260 |
| VPA | Memory Performance | 16.899 | 0.050 | 16.912 | 0.076 | -0.001 | 0.936 | 0.096 |
| VPA | Reaction time | 4.111 | 0.533 | 4.877 | 0.559 | -0.100 | 0.421 | 0.565 |
| VPA | Number of correct matches | 13.861 | 0.086 | 14.902 | 0.093 | 0.039 | 0.104 | 0.237 |
| VPA | Fluid Intelligence | 2.140 | 0.710 | 4.500 | 0.479 | -0.038 | 0.199 | 0.504 |
| VPA | Cognitive performance | 12.805 | 0.126 | 13.636 | 0.180 | 0.016 | 0.637 | 0.184 |

**Abbreviations:** MPA: Moderate-Intensity Physical Activity

VPA: Vigorous-Intensity Physical Activity

IVW: Inverse-variance weighted

**Table S3: Results from the Mendelian randomization sensitivity analysis of the association between domain-specific cognitive function and physical activity.**

| **Exposure** | **Outcome** | **Heterogeneity**  **(Cochran’s Q test)** | | | | **Pleiotropy** | | |
| --- | --- | --- | --- | --- | --- | --- | --- | --- |
|  |  | **MR-egger** | | **IVW** | | **MR-egger** | | **MR-PRESSO** |
|  |  | **Q** | **P** | **Q** | **P** | **Intercept** | **P** | **P** |
| Memory Performance | MPA | 14.970 | 0.091 | 15.182 | 0.125 | <-0.001 | 0.728 | 0.166 |
| Reaction time | MPA | 4.247 | 0.514 | 4.248 | 0.643 | <0.001 | 0.976 | 0.674 |
| Number of correct matches | MPA | 10.090 | 0.389 | 10.733 | 0.569 | 0.001 | 0.640 | 0.069 |
| Fluid Intelligence | MPA | 59.243 | 0.437 | 60.032 | 0.521 | 0.005 | 0.732 | 0.474 |
| Cognitive performance | MPA | 103.243 | 0.732 | 104.392 | 0.896 | 0.003 | 0.742 | 0.323 |
| Memory Performance | VPA | 15.643 | 0.110 | 18.134 | 0.078 | -0.006 | 0.235 | 0.078 |
| Reaction time | VPA | 8.319 | 0.139 | 10.246 | 0.114 | 0.008 | 0.330 | 0.122 |
| Number of correct matches | VPA | 7.053 | 0.133 | 7.790 | 0.168 | 0.005 | 0.553 | 0.196 |
| Fluid Intelligence | VPA | 63.429 | 0.147 | 64.239 | 0.153 | 0.004 | 0.347 | 0.439 |
| Cognitive performance | VPA | 108.432 | 0.219 | 109.336 | 0.323 | 0.002 | 0.493 | 0.231 |

**Abbreviations:** MPA: Moderate-Intensity Physical Activity

VPA: Vigorous-Intensity Physical Activity

IVW: Inverse-variance weighted


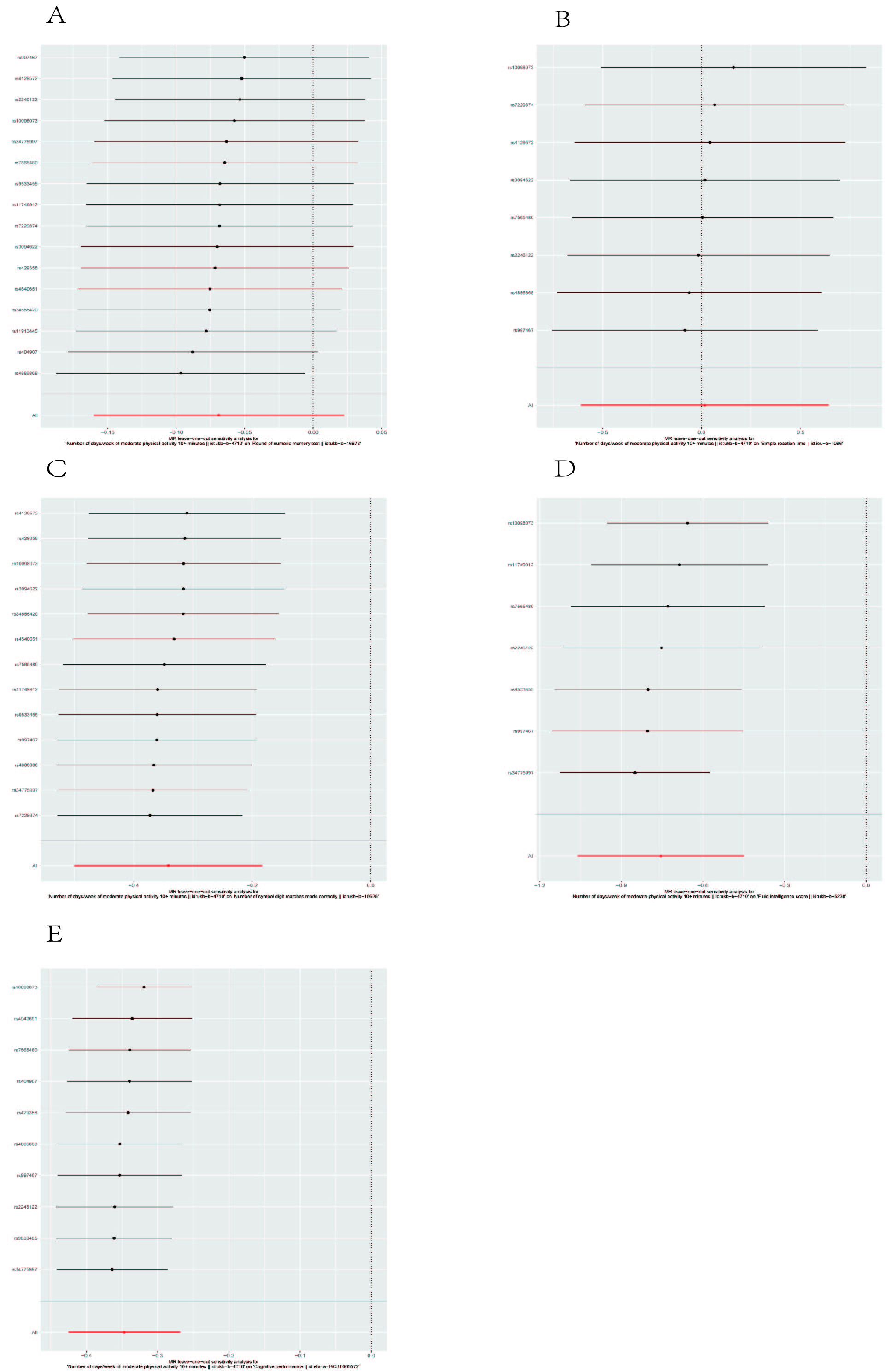


**Figure S1: Leave-one-out sensitivity analysis for the association between moderate-intensity physical activity and domain-specific cognitive function.**


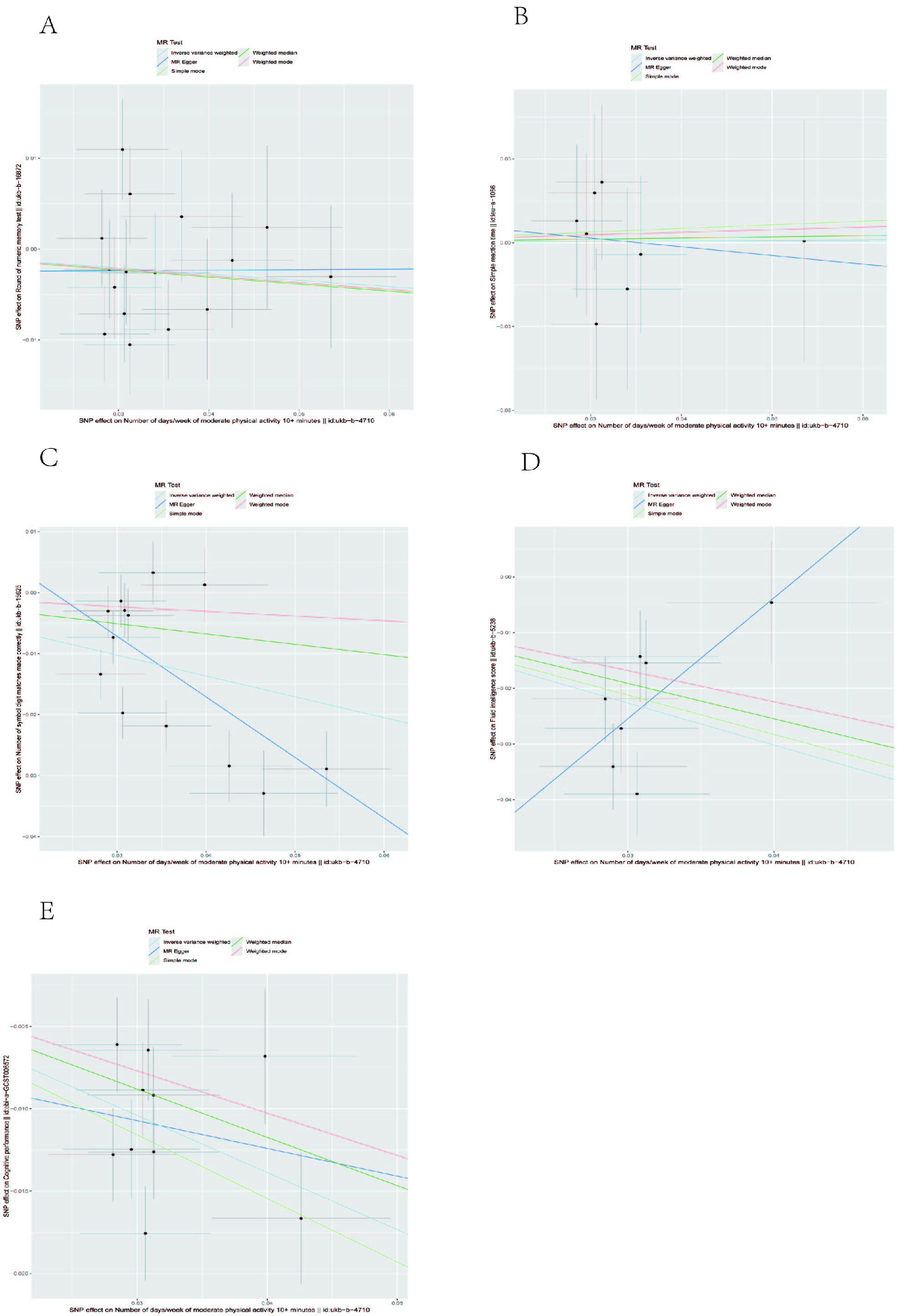


**Figure S2: Scatter plot of the genetic association between moderate-intensity physical activity and domain-specific cognitive function.**


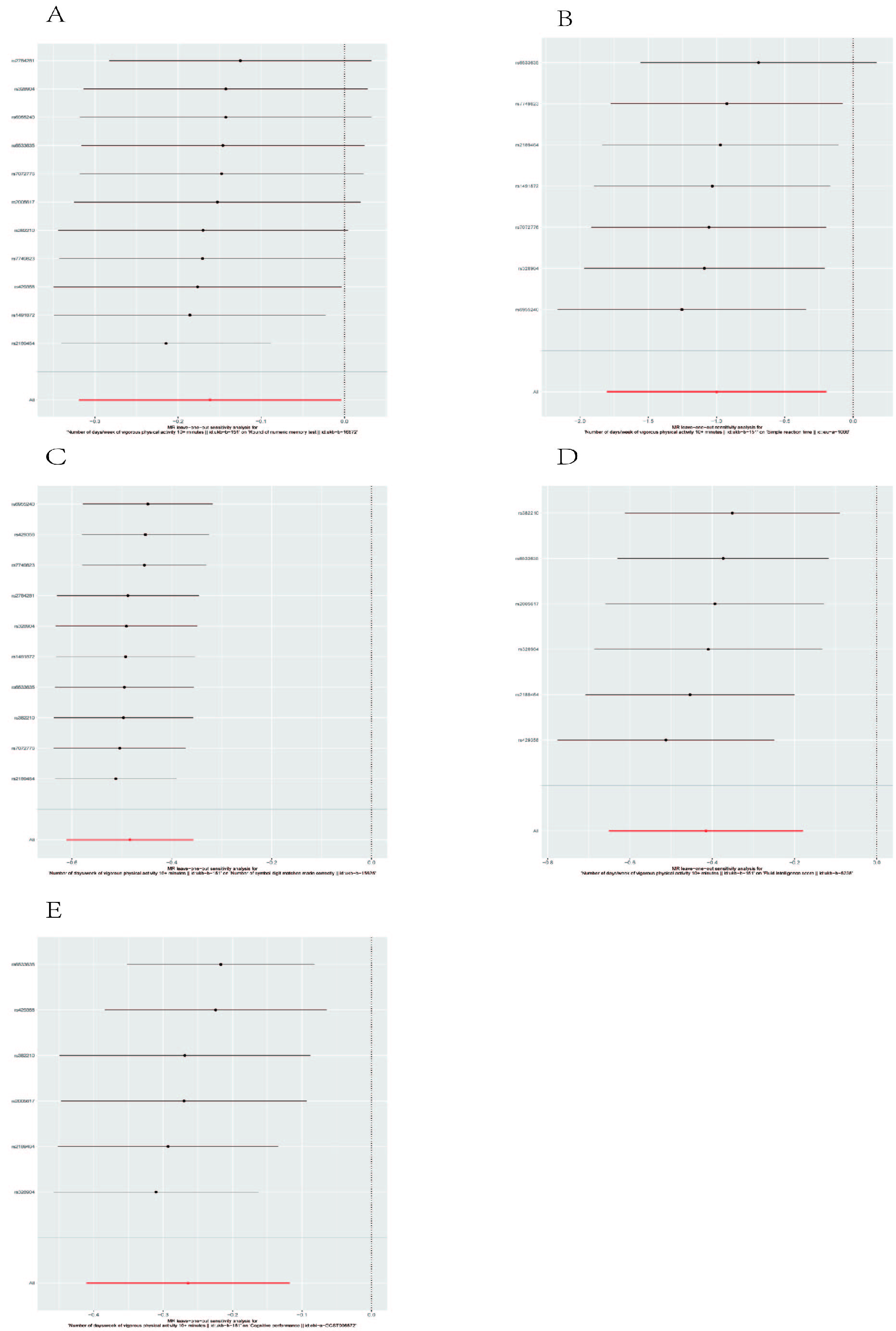


**Figure S3: Leave-one-out sensitivity analysis for the association between high-intensity physical activity and domain-specific cognitive function.**


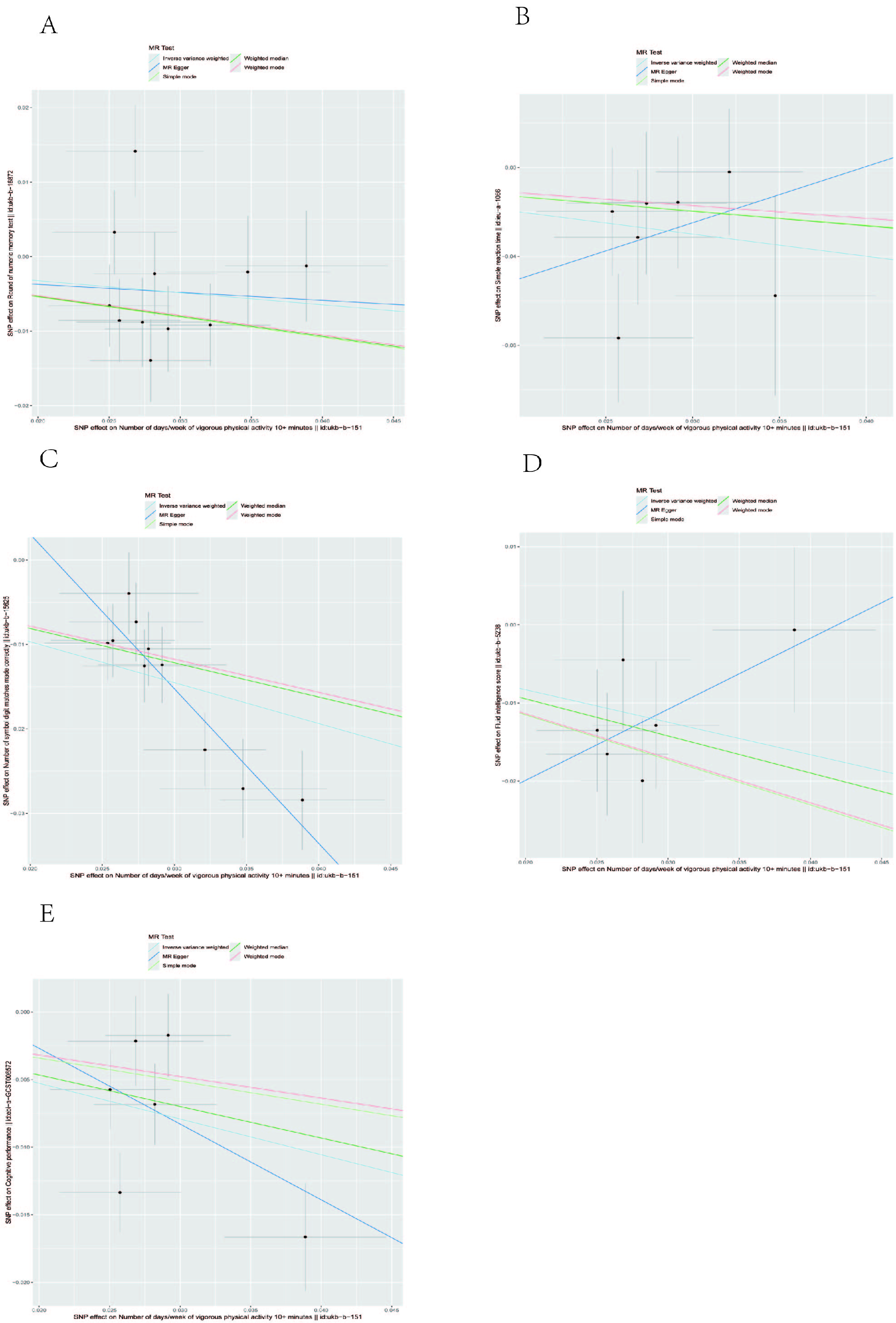


**Figure S4: Scatter plot of the genetic association between high-intensity physical activity and domain-specific cognitive function.**
